# Multiorder hydrologic Position for Europe — a Set of Features for Machine Learning and Analysis in Hydrology

**DOI:** 10.1038/s41597-022-01787-4

**Published:** 2022-10-29

**Authors:** Maximilian Nölscher, Michael Mutz, Stefan Broda

**Affiliations:** 1 Federal Institute for Geosciences and Natural Resources (BGR), Berlin, 13593 Germany; 2 Independent researcher, Berlin, Germany

**Keywords:** Hydrology, Environmental impact, Hydrogeology

## Abstract

The presented dataset EU-MOHP v013.1.1 provides multiscale information on the hydrologic position (MOHP) of a geographic point within its respective river network and catchment as gridded maps. More precisely, it comprises the three measures “divide to stream distance” (DSD) as sum of the distances to the nearest stream and catchment divide, “lateral position” (LP) as a relative measure of the position between the nearest stream and divide and “stream distance” (SD) as the distance to the nearest stream. These three measures are calculated for nine hydrologic orders to reflect different spatial scales from local to continental. Its spatial extent covers major parts of the European Economic Area (EEA39) which also largely coincides with physiographical Europe. Although there are multiple potential use cases, this dataset serves predominantly as valuable static environmental descriptor or predictor variable for hydrogeological and hydrological modelling such as mapping or forecasting tasks using machine learning. The generation of this dataset uses free open source software only and therefore can be transferred to other regions or input datasets.

## Abstract (summary)

**Table:** 

Measurement(s)	divide to stream distance • lateral position • stream distance
Technology Type(s)	remote sensing
Sample Characteristic - Environment	drainage basin • groundwatershed • catchment
Sample Characteristic - Location	Europe

## Background & Summary

In recent years, data science tools such as machine learning are increasingly applied to and specifically developed for hydro(geo)logical challenges and research questions^1,2^. In the field of hydrogeology, machine learning has been used successfully for groundwater level prediction and a variety of mapping tasks^3–13^. Since machine learning models — with the exception of hybrid- or physics-guided models — are based purely on data without any knowledge of physical processes, it is important to provide meaningful features (also called predictor or explanatory variables) that affect the target variable so that the machine learning algorithm can model the function between input and target. For surface and near-surface processes, this criterion can be more or less fulfilled by the availability of remote sensing data, whereas for modelling sub-surface processes such as in hydrogeology, this poses a serious challenge.

The key motivation for this dataset is to partially close this gap by providing a set of features that introduce hydrological context to machine learning models regarding the horizontal position of a point within its catchment. The three measures — determined by this horizontal position–are calculated for several so-called hydrological orders. Hydrologic orders represent different spatial scales, from local to regional to continental. Therefore, the measures serve as proxies for geophysical characteristics of hydrologic systems at multiple scales and complements commonly available and used features such as land-use and land-cover, geological or soil maps. This dataset is strongly inspired by Belitz *et al*.^14^ and adapts their ideas and methods to the “EU-Hydro - River Network Database”^15^ but — in contrast — using free open-source software and a strong focus on reproducibility. This concept could be spatially further extended by applying the presented methods to global river network or hydrograph datasets, such as HYDRO1k^16^ or MERIT Hydro-Vector^17^. For more detailed background on the concept and methods, we refer to Belitz *et al*.^14^.

In their study, Belitz *et al*.^14^ also provide results from case studies to prove that the multiorder hydrologic position is a valuable feature when mapping diverse geophysical target variables using machine learning. Its benefit to the performance of machine learning models has also been acknowledged by several other studies^7,18,19^.

The gridded maps of the EU-MOHP dataset^20^ reflect a static geophysical attribute and can be used as features for machine learning or general modelling tasks in the field of hydrology and hydrogeology. As is generally the case in the geosciences, “static” in the sense of time-invariant is strongly relative, because river networks also change over time, but rather slowly compared to groundwater level fluctuations. This dataset can be applied at multiple spatial scales — from local through regional to continental scales. Examples of use cases can be the mapping of hydrogeochemical parameters or hydraulic variables, the prediction of groundwater levels or catchment classification tasks using unsupervised machine learning methods. But it can also be used for exploratory data analysis.

The EU-MOHP v013.1.1 dataset^20^ comprises the three measures

**Fig. 1:**
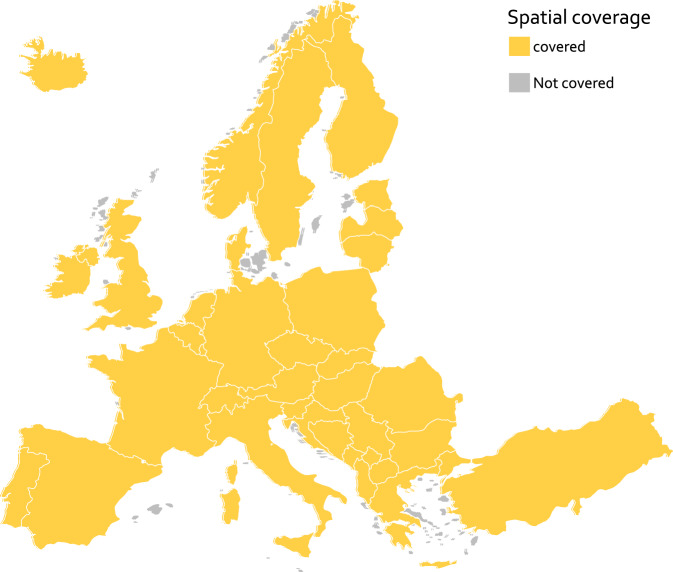
Spatial coverage of the dataset which is determined by the study area data layer.

- divide to stream distance (DSD),
- lateral position (LP) and
- stream distance (SD).

for each hydrologic order. This results in ${n}_{measures}\cdot {n}_{hydrologicorders}=3\cdot 9=27$ different metrics to be used as features. Spatially, the dataset covers major parts of physiographical Europe and all of the 39 countries in the European Economic Area (EEA39). More precisely, it covers the 10 largest contiguous land masses of the EEA39 (Fig. 1).

Conceptually, the three measures DSD, LP and SD of EU-MOHP^20^ are based on the idea that the location in the hydrologic systems matters^14^. A location can be e.g. close to the confluence of two large rivers or in another extreme be close to the catchment boundary of headwater streams. Such differences in the location in the hydrologic context contain valuable information for models as they determine a major part of the dynamics of the system, e.g. recharge, discharge, fluctuations or the temporal delay to input signals like meteorological forcings. The location or hydrologic position in this case refers to the position of a point between the nearest river and its catchment boundary. Thiessen divides are used as catchment boundaries instead of divides that are generated from digital elevation models (DEM) for a variety of practical reasons as described in Belitz *et al*.^14^. For further details on Thiessen divides, see section Methods.

Based on the river network and the Thiessen divides, the EU-MOHP^20^ measures are calculated with

1 $${{\rm{DSD}}}_{i}={{\rm{DS}}}_{i}+{{\rm{DD}}}_{i}$$

2 $${{\rm{LP}}}_{i}=\frac{{{\rm{DS}}}_{i}}{{{\rm{DS}}}_{i}+{{\rm{DD}}}_{i}}$$

3 $${{\rm{SD}}}_{i}={{\rm{DS}}}_{i}$$

where DS_*i*_ is the distance to the nearest stream, coast or surface water body of the hydrologic order *i* and DD_*i*_ is the distance to the nearest divide of the hydrologic order *i*. The terms “river” and “stream” are used interchangeably here, but stream refers more to the digitial representation of a river.

**Fig. 2:**
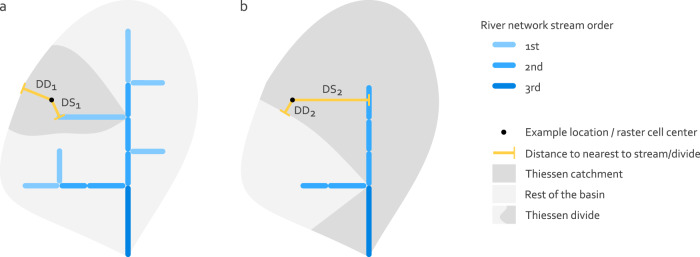
Schematic representation of MOHP measures using two examples for the hydrologic orders 1 (**a**) and 2 (**b**). DS is the horizontal distance to the nearest stream and DD is the horizontal distance to the nearest Thiessen divide under the condition that the divide is on the same side of the stream as the raster cell center (black point).

These three measures are not only calculated for a single scale, but are transferred to several scales through the second important part of the concept, the previously mentioned hydrological orders. This is particularly valuable because the importance of the various hydrological processes depends on the scale. It therefore allows both investigations at different scales and consideration of different depths, as the depth of groundwater flow paths generally increases with greater hydrological scale. The hydrologic orders are based on the stream orders of the river network. For a specific hydrologic order *i*, only streams with a stream order > = *i* are used, whereas those with stream order <*i* are removed (e.g. for hydrologic order 2, all streams with stream order 2, 3, 4 and greater are used; compare Fig. 2a,b). This can also be understood as a step-wise pruning of the smallest streams from the river network for each hydrologic order, which subsequently represent different spatial scales. Here, the stream orders are defined according to Strahler (1957)^21^ where all streams between the headwaters and the first confluence are assigned to the first stream order. The stream order downstream of a confluence increases by 1 if the upstream stream orders are equal. If the stream orders are not equal, it inherits the greater stream order of the confluent streams.

**Fig. 3:**
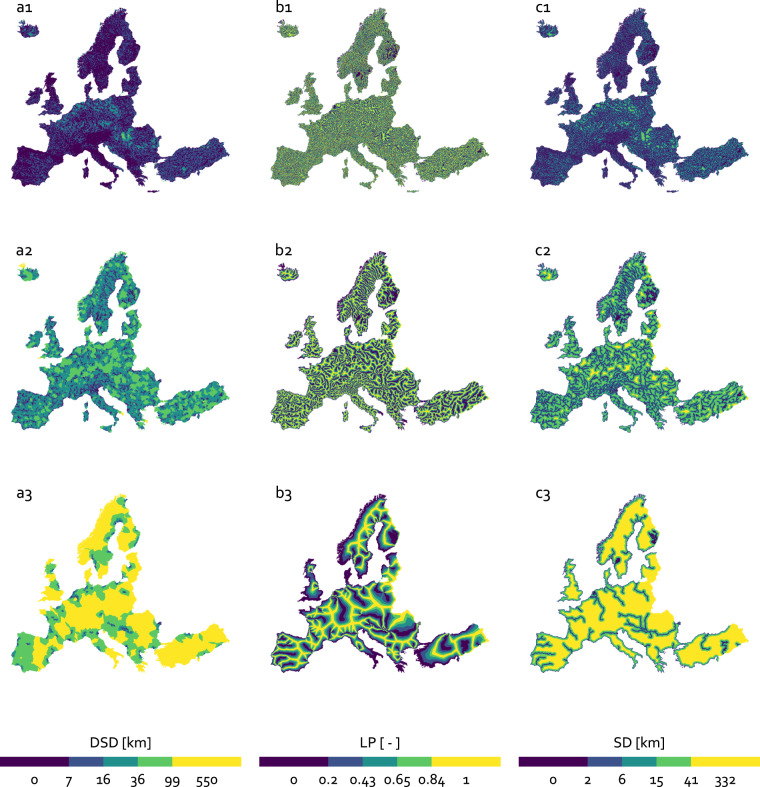
Resulting maps of the three EU-MOHP measures DSD (**a**), LP (**b**), and SD (**c**) in the columns exemplary for the three hydrologic orders 3 (1), 5 (2) and 7 (3) in the rows. Note that the breaks of the binned colour scale is based on quantiles.

Figure 3 shows the resulting EU-MOHP v013.1.1^20^ exemplary for the three hydrologic orders 3, 5 and 7 as maps.

## Methods

### Underlying dataset

The generation of this dataset is based on two datasets, first the “EU-Hydro–River Network Database” version v013^15^ and “EU-Hydro–Coastline” version v013^22^ with the advantage that data dependencies are low. From these two datasets, the four data layers (1) river network, (2) surface water bodies, (3) river basins/study area and 4) coastline were derived (see Table 1). Due to this relatively low input data requirements, it is possible to transfer the presented methodology to other regions or datasets with only little effort.

**Table 1:** Overview of the required input data.

No Data layer	Data source	Layer in .gpkg files	Geometry type	Description
1 river network	EU-Hydro–River Network Database	River_Net_l	linestring	representing stream lines of rivers
2 surface water bodies	EU-Hydro–River Network Database	InlandWater	polygon	representing lakes, ponds and wide rivers
3 river basins/ study area	EU-Hydro–River Network Database	_eudem2_basins_h1	polygon	representing the river basins
4 coastline	EU-Hydro–Coastline	-	linestring	representing the coastline

**Fig. 4:**
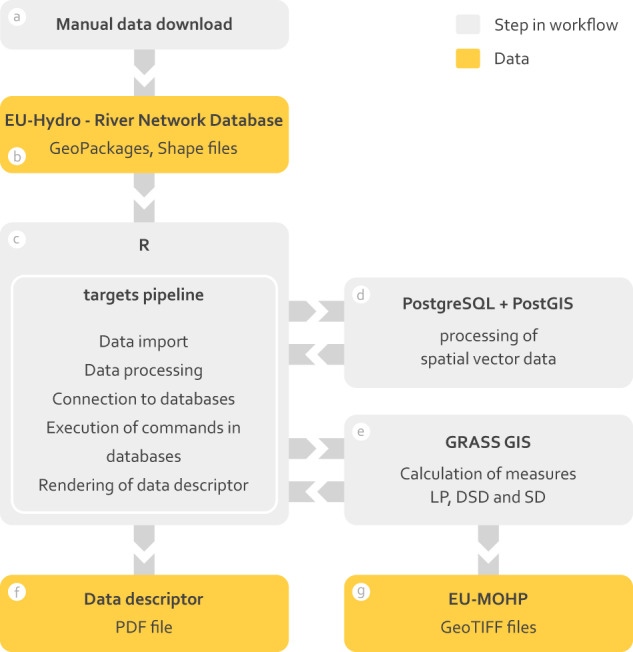
Workflow of the data processing in different software.

The “EU-Hydro–River Network Database”^15^ as well as the “EU-Hydro–Coastline”^22^ has been manually downloaded from the Copernicus - Land Monitoring Service website (see Fig. 4a). The river network data is split into two GeoPackage (*.gpkg*) files for each of the 35 major river basins in the EEA39 countries, one with the naming scheme “*drainage_network_*<*river name*>*_public_beta_v009.gpkg*” and the second with “*euhydro_<river name>_v011.gpkg*”. The coastline data is stored in a single Shapefile (*.shp*) file (see Fig. 4b). All files have a total size of approximately 14 GB when unzipped.

The single *.shp* file containing the coastline has a size of 288 MB. For instructions on accessing this underlying data, see Usage Notes.

### Processing

The generation of the presented dataset requires several computationally expensive processing steps. For this reason and to make the methods more reproducible and maintainable, all processing steps are executed and controlled by a processing pipeline in the R programming language using the *targets* package (Fig. 4c)^23,24^. This processing or *targets* pipeline can be seen as programming script tracking changes in the source code and the data with the major advantage among many that it can skip processing steps that are still up-to-date and re-executes those that need to be updated. Due to the large memory size requirements for this dataset as well as for computational speed reasons, a PostgreSQL database with the PostGIS extension is used for certain processing steps of vector data and a GRASS GIS database is used for all final raster-based calculations of the EU-MOHP^20^ metrics (Fig. 4d,e). The calculations in the databases are also tracked and executed by the processing pipeline. In the following, the relevant steps of the methods are described. For a fully comprehensive description of all details, we refer to the source code itself (see Code availability).

### Data preparation

In the following, the most relevant processing steps are described. These steps are part of the previously described pipeline and are defined as so-called targets in the source code of the pipeline. To simplify the description, the processing steps are grouped here according to the previously mentioned data layers.

#### Study area

The preprocessing steps to define and generate the study area are described first because it is required for the processing of all other data layers. The study area also defines the spatial coverage of the final product. For the generation of the study area, the layer *_eudem2_basins_h1* in the previously mentioned GeoPackage file with the naming scheme containing the suffix “*drainage_network*” (see Table 1) is used. It contains polygon geometries representing sub-basins of the major the river basins. Firstly, all polygon geometries belonging to European oversea territories such as the French islands in the Caribbean are removed. Then, the remaining polygons are merged. Subsequently, out of these polygons of contiguous land masses the 10 largest polygons by area are chosen as study area.

#### River network

The river or hydrographic network is based on the linestring geometries from the layer *River_Net_l* in the previously mentioned GeoPackage file with the naming scheme containing the suffix “*euhydro*” (see Table 1). This data layer requires more processings steps than the other three data layers. Firstly, specific linestring geometries are removed from the river network. These linestrings comprise all geometries categorized as canal or ditch in the attribute column *dfdd* encoded with the values BH020 for canal and BH030 for ditch^25^. These are mainly removed for the following two reasons: Firstly, many of the canal and ditch geometries have missing stream order values, which is required for the following processing steps and secondly, it is assumed that canals are often hydraulically disconnected from the natural hydrological system because of their impermeable side walls and canal bed. Besides this, the overall importance of canals and ditches is low when comparing their number of geometries to the number of river geometries (difference of three orders of magnitude). Furthermore, all linestring geometries categorized as non-perennial rivers in the attribute column *hyp* encoded with the values 2 (intermittent), 3 (ephemeral) and 4 (dry) are removed^25^. After this filtering, more than 1.05 million geometries remain. Then, missing and invalid stream order values are imputed with the value 1 as first stream order. This ensures that related geometries are at least included in the first hydrologic order. Subsequently, the river network geometries are clipped to the study area.

**Fig. 5:**
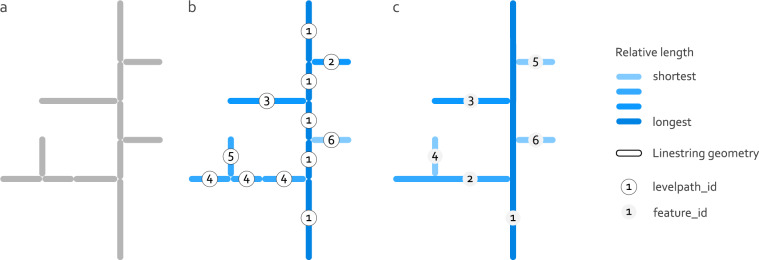
Schematic representation of the river network and its linestring geometries before generating the mainstems (**a**), after the identification of mainstems including the column levelpath_id (**b**) and after merging the linestring geometries by column levelpath_id and adding a feature_id column (**c**).

The next essential processing step implements a method to obtain linestring geometries that represent the mainstems of the river networks as described in the Supplementary of Belitz et. al. (2019). A mainstem is defined here as the longest path from the head water to the next most distant river mouth (see geometries with the same *levelpath_id* in Fig. 5b). In Fig. 5b the concept mainstems is schematically shown. In this figure, a mainstem consists of linestring geometries with the same *levelpath_id*. Belitz *et al*.^14^ made use of the column “LevelPathID” in their underlying NHDPlusV2 river network dataset^26,27^. As a comparable column does not exist in the “EU-Hydro–River Network Database” dataset^15^, its generation is a required preprocessing step. This step is especially essential when applying these methods to river network data that does not provide suitable columns to generate the mainstems from. The generation of this required column *levelpath_id* for the river network dataset^15^ involves the following steps. Firstly, a river network is derived separately for each hydrologic order by keeping only geometries with a stream order equal or greater than the specific hydrologic order as described in Background & Summary (see also Fig. 2). The following steps are repeated for each hydrologic order. The river network is sorted by the column *longpath* in descending order. The column *longpath* indicates the length of the path from the start node of a linestring geometry to the end node of the most downstream geometry of the river network. Then, starting with the top geometry, all line geometries are determined that are connected with each other by means of the columns *object_id* and *nextdownid*.The column *object_id* provides an unique ID for every linestring geometry and *nextdownid* indicates the *object_id* of the next downstream geometry. The now identified linestrings constitute the longest mainstem and are removed from the original river network. This is now iteratively repeated for the second top linestring in the remaining river network and so on.

Subsequently, the column *levelpath_id* is added as a unique ID for all geometries belonging to the same mainstem (Fig. 5b). The geometries of the respective river network are then merged based on this column (see difference in linestring geometries between Fig. 5b,c). This results in a river network for each hydrologic order separately with a reduced number of geometries as multiple geometries are now summarised into mainstems.

**Fig. 6:**
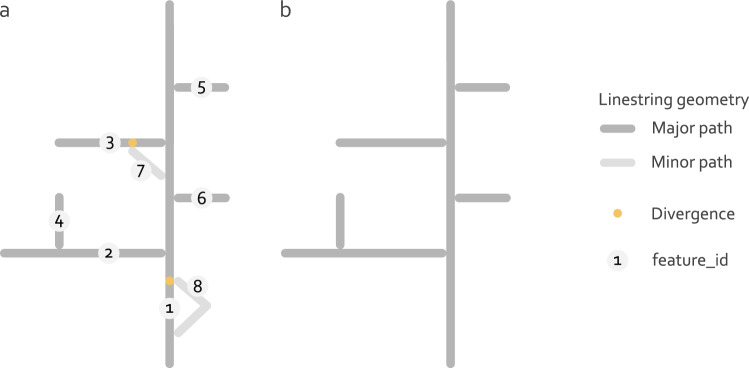
Schematic representation of the river network and its linestring geometries including divergences before (**a**) and after (**b**) the removal of minor paths. The linestring geometry with the feature_ids 7 and 8 have been removed from the river network in B, because they intersect other linestring geometries with both, the start and end node.

The next step addresses the occurrence of flow splits in the river network. A flow split or divergence is defined here as junction of linestring geometries with more than one linestring geometry representing out-flowing streams (orange marks in Fig. 6). To transfer the methods from Belitz *et al*.^14^ for the calculation of EU-MOHP^20^, it is required to remove minor flow paths that originate from such divergences from the river network. A classification of linestring geometries into major and minor flow paths is not directly provided by any column in the underlying river network dataset. Belitz *et al*.^14^ used the column *divergence* for removing all minor flow paths. Here, this is achieved by removing all linestring geometries that intersect other linestrings with both the end and start node. The removal of these minor flow paths is not done for the first hydrologic order to include all linestrings in at least one order. The implementation of these steps pointed out errors in the river network dataset^15^. These errors are related to errors of values in the columns *longpath* and *nextdownid*. Based on visual inspection, they occur in the french river networks of Garonne, Loire and Seine and are corrected programmatically during processing.

Then, the river networks are sorted by the length of the linestring geometries in descending order and provided with an unique ID for each geometry in the column *feature_id* (see *feature_id* in Fig. 5c).

#### Surface water bodies

The surface water bodies are derived from the layer *InlandWater* in the GeoPackage file with the naming scheme containing the suffix “*euhydro*” (see Table 1). A filter is applied to retain only the geometries of surface water bodies that have an area greater than four times the area of the grid cell. Another filter is applied to remove all geometries that do not intersect with the river network geometries. Since the river networks of the 9 hydrologic orders differ from each other, this second filter is applied individually for each of the river networks. This results in a dataset of surface water bodies for each hydrologic order.

#### Coastline

The data layer coastline is derived from the Shape file related to the “EU-Hydro–Coastline” dataset^22^ (see Table 1). Like rivers, the ocean, defined by the coastline, is an area where water accumulates and therefore its spatial representation is necessary for the generation of this dataset^14^.

**Fig. 7:**
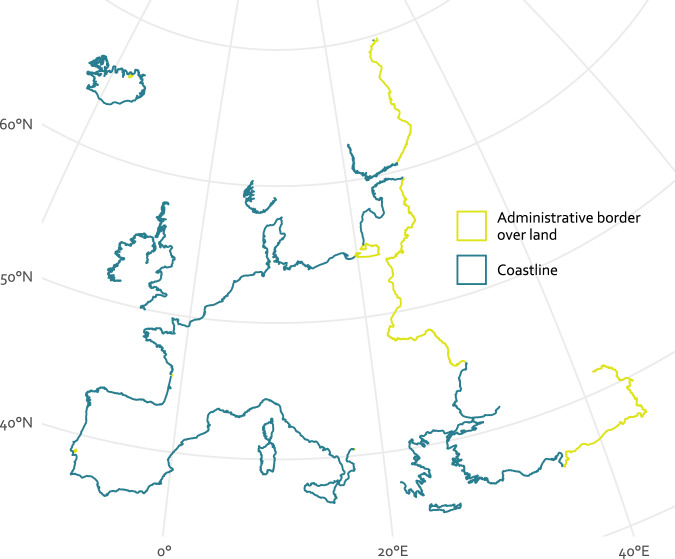
Map showing location and the spatial distribution of coastline and administrative borders over land resulting from the preprocessing.

Firstly, the polygon geometries of the underlying Shape file are merged. Then, a buffer of 3000 m is added to the merged geometries. This is necessary to ensure that the outline of the study area intersects with the coastline polygon geometries for the next step. Without this buffer, discrepancies between the study area and the coastline can be noticed. These discrepancies would lead to undesired results after the next step. The value of 3000 m is derived from visual inspection. The resulting multipolygon geometries are intersected with the outline of the study area to obtain the coastline as linestring. Those parts of the study area that do not intersect with the polygon geometries are categorized as “administrative borders over land”. This intersection then ensures that the coastline exactly aligns with the study area outline. The resulting coastline is shown in Fig. 7. The coastline is then added to each river network of all hydrologic orders.

### EU-MOHP

After obtaining all four required data layers as described previously, the next and last processing step comprises multiple smaller steps with the final goal to calculate and export the EU-MOHP^20^ metrics. Because the processing is analogous for all hydrologic orders and all of the 10 polygon geometries of the study area, this step is described only once in general terms. As all processing steps described below require grid based computations, a GRASS GIS database is used (see Fig. 4e).

The four data layers study area, river network including the coastline and surface water bodies of the respective hydrologic order and the coastline are written into the GRASS GIS database. The projection of the GRASS GIS database is set to the ETRS89 Lambert Azimuthal Equal-Area projection coordinate reference system (EPSG: 3035). The spatial resolution of the raster cells is set to 30 m.

#### Thiessen catchments and distance to stream (DS)

As described in Background & Summary, the catchment boundaries are required to determine DD (see Eqs. (1, 2) or Fig. 2). Therefore, Thiessen divides are used. A Thiessen divide is the outline of a Thiessen catchment which in turn is the area containing all points in a river network to which a river is closer than any other river^28^. One major advantage is that Thiessen divides can be calculated purely based on the river network itself while avoiding issues such as closed lows in the resulting metrics^14^. This advantage outweighs the numerous minor problems associated with DEM-based catchments, especially when taking into account the uncertain correspondence of the subsurface catchment to the surface catchment. A detailed discussion on the preference of Thiessen divides over topographic divides is provided in Belitz et. al. (2019), section 2.2.0^14^. In order to obtain Thiessen divides, the first step is to calculate the euclidean distance from each raster cell center to the nearest river network geometry. The resulting distances correspond to DS in Eqs. (1–3) or Fig. 2). This step also determines the feature ID of the nearest geometry for all raster cells. Then, the polygons representing Thiessen catchments are derived by merging all raster cells that are assigned to the same feature ID. Finally, the outlines of these polygons are used as Thiessen divides.

#### Distance to divide (DD)

To obtain the distance to divide (DD) for each raster cell, the distance from each raster cell center to the nearest Thiessen divide is calculated. But the determination of the nearest Thiessen divide cannot be achieved by a simple nearest neighbour search as it is used for the calculation of DS and the feature ID of the nearest river. Implementing the physical reality that in catchments the water accumulates and runs off in rivers requires an additional condition. This condition has to ensure that distances to the nearest divide are not calculated across rivers. In other words the nearest Thiessen divide for each raster cell must not lie on the other side of the river. In other words, when drawing a imaginary line between the nearest Thiessen divide and the grid cell center, this line must not cross a river geometry (see black line versus red line in Fig. 13). Without this condition the geometric center line of the Thiessen catchments would be considered as areas of accumulation and discharge. To meet this condition, the GRASS GIS command r.walk was used. Minor inaccuracies regarding this command for the described purpose are noted in Technical Validation. The calculated distances correspond to DD in Eqs. (1, 2) or Fig. 2.

#### Measures DSD, LP and SD

Based on the two calculated raster layers containing the distances DS and DD, the three EU-MOHP measures DSD, LP and SD are now calculated by the application of the equations Eqs. (1–3) and the GRASS GIS raster map calculator (“r.mapcalc”). In order to reduce the storage size, the raster values of the measure LP are multiplied by a factor of 10,000 and rounded to be able to store them as integer values with two decimal digits. The two measures DSD and LP are rounded to the nearest integer. Finally, the resulting raster layers for LP, DSD and SD are exported from the GRASS GIS database and stored on disk as GeoTIFF files with*.tif* file extension.

### Data descriptor

To enhance the reproducibility of the data descriptor manuscript itself, it is generated as part of the processing pipeline. Also all tables and all data derived figures are created from within the pipeline. This ensures that all figures are up-to-date and reflect the most recent state of the methods. The descriptor is written in RMarkdown from which a LaTeX and a PDF file are generated using the *knitr* package^29,30^.

## Data Records

The presented EU-MOHP v013.1.1 dataset^20^ is available on the Hydroshare repository at 10.4211/hs.0d6999591fb048cab5ab71fcb690eadb. The dataset represents gridded maps with a spatial resolution of 30 m. It is divided into multiple GeoTIFF files with a*.tif* file extension. Each file represents data on one of the three EU-MOHP^20^ measures–LP, DSD, and SD–for one hydrologic order for a different study area polygon (spatial coverage). The file names are structured according to the file naming scheme “*mohp_europe_<region name for spatial coverage>_<abbreviation of the EU-MOHP measure>_<hydrologic order>_<spatial resolution>.tif*”. The placeholders including “*<*” and “*>*” can be theoretically replaced by any combination of the values summarized in Table 2. But not all study area polygons have a river network for each hydrologic order. For example, the study area polygon for the island of Sardinia only has rivers up to a maximum streamorder of 6 and therefore only a maximum hydrologic order of 6. This means that there are no GeoTIFF files for Sardinia for hydrologic orders 7–9. Therefore, the total number of files is ${n}_{measures}\cdot {\sum }_{i=1}^{{n}_{hydrologicorders}}{n}_{studyareapolygons,i}=3\cdot {\sum }_{i=1}^{9}{n}_{studyareapolygons,i}=192$.

**Table 2:** Overview of the output file naming scheme and its placeholder values of the EU-MOHP dataset.

Placeholder in output file name	Value	Description
<region name for spatial coverage>	europemainland	Raster data covers the contiguous land area of continental Europe,…
	finland-norway-sweden	…the Scandinavian countries Finland, Norway and Sweden
	france	…Corsica
	greece	…Creta
	iceland	…Iceland
	italy1	…Sicily
	italy2	…Sardinia
	turkey	…Turkey
	unitedkingdom	…United Kingdom
	unitedkingdom-ireland	Ireland and Northern Ireland
<abbreviation of the EU-MOHP measure>	dsd	Divide to stream distance
	lp	Lateral Position
	sd	Stream distance
<hydrologic order>	hydrologicorder1	Hydrologic order (increasing order translates to larger catchments and therefore a larger scale)
	hydrologicorder2	
	hydrologicorder3	
	hydrologicorder4	
	hydrologicorder5	
	hydrologicorder6	
	hydrologicorder7	
	hydrologicorder8	
	hydrologicorder9	
<spatial resolution>	30m	Spatial resolution

Files for any combination of the placeholder values exists except for those study area polygons (<region name for spatial coverage>) that have no streams for certain hydrologic orders. The values are inserted for the respective placeholder in “mohp_europe_<region name for spatial coverage>_<abbreviation of the EU-MOHP measure>_<hydrologic order>_<spatial resolution>.tif”. For example, selecting the first value of each placeholder results in the file name “mohp_europe_europemainland_dsd_hydrologicorder1_30 m.tif”. The spatial coverage of the values for “<region name for spatial coverage>” is shown in the mentioned interactive map in the Github repository.

The GeoTIFF files derived in section Measures DSD, LP and SD, were uploaded to Hydroshare as separately compressed files with the file extension *0.7z* using the free and open-source file archiver program 7-Zip. Each *0.7z* file corresponds to one *.tif* file.

On Hydroshare you have the option to either select all *0.7z* files and download them as a zipped bagit archive or download a custom selection of files if your are only interested in a specific region (area of interest) or specific hydrologic orders. For creating a user defined selection you can use the search bar to filter the files for a spatial coverage or a hydrologic order as described on Hydroshare website of this dataset. If you want to check more precisely whether your area of interest is covered by this dataset at all or which files are relevant, please see the interactive map on Github (https://mxnl.github.io/macro_mohp_feature/).

The presented EU-MOHP dataset^20^ has version v013.1.1 The version is generated as a composition of the “EU-Hydro–River Network Database”^15^ version (v013) and a major and a minor version number (1.0) that are related to the methods of this dataset.

## Technical Validation

### Statistical summary

The EU-MOHP dataset^20^ consists of calculated values based on a hydrological concept and therefore cannot be validated by observations or measurements. As a first approximation, a statistical summary based on a sample of every 100th grid cell per row and column is used for validation. Table 3 provides the median, mean, minimum and maximum value of the three measures across all hydrologic orders. In accordance with the theoretical background, the values of mean, median and max of DSD and SD are increasing with increasing hydrologic order (see also Fig. 3a1-3 and c1-3). This also highlights the different spatial scales. This increase is not shown by median or mean values of LP due to the fact that LP is a relative measure. The minimum and maximum values of LP are 0 and 1 as expected across all hydrologic orders. The only anomaly here are the median and mean related to the ninth hydrologic order. These lower values compared to all other hydrologic orders are related to the spatially highly unequal distribution of the river network in this case in combination with the shape of the coastline of Europe. This will be discussed in next paragraph. Another anomaly are the minimum values of DSD at higher hydrologic orders. Their deviation from 0 is caused by the decreasing probability that a grid cell center lies exactly on the intersection of a river and divide at higher hydrologic order.

**Table 3:** Statistical summary of the calculated measures DSD, LP and SD across all hydrologic orders.

Hydrologic order	DSD [km]				LP [-]				SD [km]			
	min	median	mean	max	min	median	mean	max	min	median	mean	max
1	0.00	1.56	1.99	76.20	0	0.54	0.52	1	0	0.72	1.07	42.79
2	0.00	3.21	3.73	76.20	0	0.57	0.54	1	0	1.57	2.08	42.79
3	0.00	6.43	7.24	89.06	0	0.56	0.54	1	0	3.08	3.97	46.12
4	0.00	13.08	14.46	200.67	0	0.55	0.53	1	0	6.13	7.69	54.55
5	0.00	26.59	28.78	216.38	0	0.54	0.52	1	0	12.12	14.96	100.04
6	0.00	56.54	61.51	425.58	0	0.55	0.52	1	0	25.36	30.99	193.41
7	0.00	117.59	128.93	549.84	0	0.53	0.51	1	0	50.23	62.33	331.55
8	0.28	233.22	247.53	864.88	0	0.50	0.50	1	0	91.80	112.63	419.32
9	0.29	763.36	919.80	2531.84	0	0.17	0.28	1	0	129.08	159.89	564.59

**Fig. 8:**
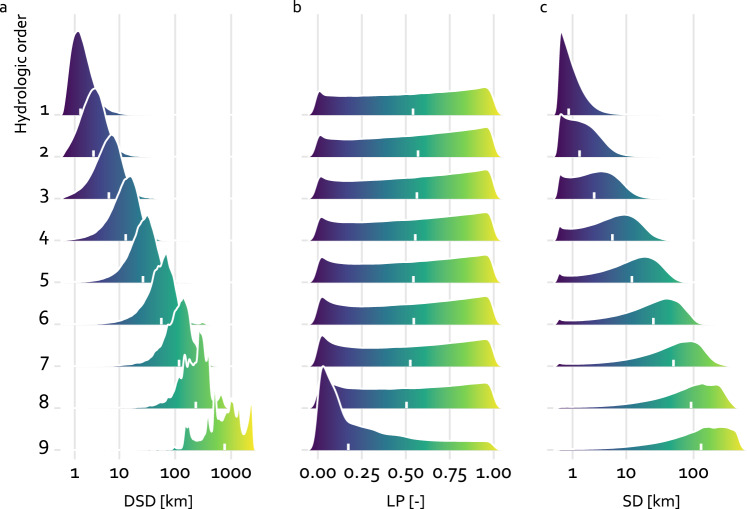
Ridgelines showing the distribution of the three measures DSD (**a**), LP (**b**) and SD (**c**) for all nine hydrologic orders. The white tick mark represents the median.

For a more comprehensive overview of the distribution of the values of the three measures, Fig. 8 shows the density of the values for all hydrologic orders. Here, the overall increase of the values of DSD and SD with increasing hydrologic order as previously seen in the Table 3 is clearly apparent. Further, the distribution of DSD values changes from a left-skewed uni-modal distribution (1st hydrologic order) to a multi-modal distribution (9th hydrologic order). This change of mode is caused by the many peninsulas of different sizes of the European coastline. Its shape has many peninsulas of different sizes. Examples for such peninsulas ordered from smaller to bigger are Denmark, Bretagne, Greece, Italy and the Iberian Peninsula. With increasing hydrologic order the number of rivers in these peninsulas reduces. If there is no river present anymore, the distribution of DSD show a peak at values related to this peninsula. Belitz *et al*.^14^ referred to this effect as peninsula effect. This also explains the evident change of the distribution of LP of the 9th hydrologic order compared to all other orders.

**Fig. 9:**
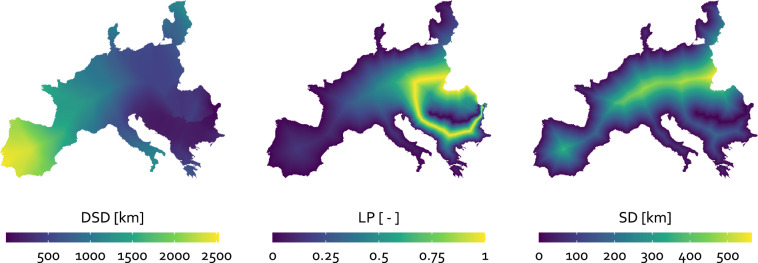
Resulting maps of the three EU-MOHP measures DSD, LP and SD (from left to right) for the 9th hydrologic order.

This effect is most pronounced in the 9th hydrologic order, where the last few hundred kilometers of the Danube river before its river mouth into the Black Sea is the only river segment in the whole of continental Europe (Fig. 9). The utilization of this dataset at locations with such an effect is very limited at best.

### Cross-comparison with original MOHP methods

**Fig. 10:**
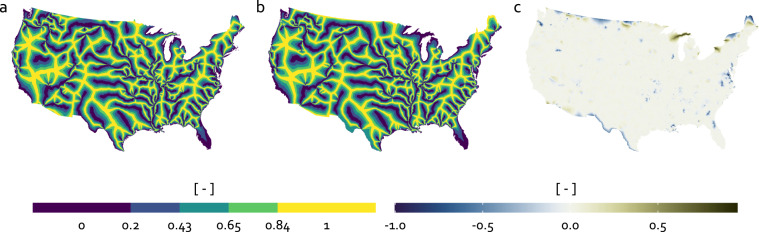
Maps showing the Original LP7 (**a**), the Reproduced LP7 (**b**) and the absolute difference between Original LP7 and the Reproduced LP7 (**c**) for the contiguous US.

To further assess the quality of the applied methodology in this study, a cross-comparison with the original MOHP dataset for the contiguous US by Belitz *et al*.^14^ was performed. Therefore, we reproduced parts of the original MOHP dataset by applying our methodology to the NHDPlusV2 dataset^26^, which is the underlying dataset of the original MOHP dataset, and compared these reproduced results to the original dataset^14^. As the methodology is analogous for all hydrologic orders and the values of all three measures (DSD, LP and SD) have the same dependencies (DD and DS), it is sufficient to cross-compare LP for a single hydrologic order. For visual purposes, the 7th hydrologic order was selected. Accordingly, the reproduced dataset will be referred to as “Reproduced LP7” and the original as “Original LP7”. Figure 10 shows a side-by-side comparison between the Original LP7 (a) and Reproduced LP7 (b). From visual inspection, the major patterns appear very similar on both maps. Differences can be mainly observed in proximity to the administrative borders to Canada and Mexico. These difference among some other minor ones are due to deviations from the original methodology. Although the methodology of the original MOHP dataset^14^ is generally well described, it was not possible to fully comprehend and reproduce all steps due to the source code not being publicly available. For this reason, the coastline used for the cross-comparison is not fully identical to the coastline used for the original dataset. Likewise, neither river networks from the two neighboring countries Canada and Mexico nor surface water bodies in general are included in the reproduced dataset. The regions most affected by these differences are excluded from the quantitative cross-comparison. Figure 10c shows the absolute difference between both maps from Fig. 10a,b defined as

4 $${\rm{Absolute}}\;{\rm{difference}}=\frac{{\rm{Original}}\;{\rm{LP}}7-{\rm{Reproduced}}\;{\rm{LP}}7}{10,000}.$$

**Fig. 11:**
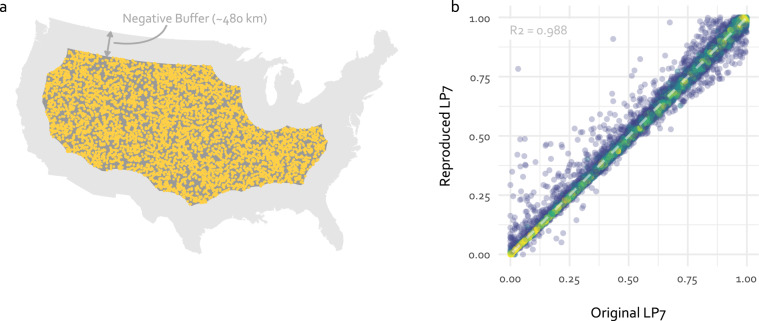
(**a**) Sampling strategy for the quantitative cross-comparison. The sampling locations are shown in yellow. Due to aesthetic reasons, only half of the total 10,000 points are shown here. (**b**) Raster cell values at the sampling points for the Original LP7 and Reproduced LP7. The point colour represents point density with yellow for high to blue for low density.

The division by 10,000 is applied to rescale the values to a range from 0 to 1. In this figure, the previously described differences along the borders and close to surface water bodies become more visible. This figure also shows that the values of the absolute difference is predominantly close to 0 (greyish colour) across all of contiguous US indicating no or small differences. In addition to this visual comparison, a quantitative cross-comparison is performed by comparing the raster cell values of the Original LP7 and the Reproduced LP7 at 10,000 randomly distributed points. To account for these expected discrepancies between the reproduced and original datasets near the coast and at administrative boundaries over land, a negative buffer of 300 miles (about 480 km) inland was used to exclude these regions from the quantitative cross-comparison. Figure 11a schematically shows the sampling strategy including the location of half of all 10,000 sampling points.

Figure 11b shows the raster cell values of the Original LP7 and the Reproduced LP7 at the sampling locations. While a small proportion of all points is distant from the dashed equal-value line, the vast majority is close, indicating a the Original LP7 values are well reproduced. To quantify this, a linear regression model was applied to all points. The *R*^2^ of the fitted model is 0.988. In summary, the cross-comparison shows a very good agreement of the methodology used in this study with the described methods in Belitz *et al*. (2019). The largest differences in the results can be explained by deviations in the reproduced methodology, as already mentioned (river networks in neighbouring countries, surface water bodies).

### Underlying river network dataset

**Fig. 12:**
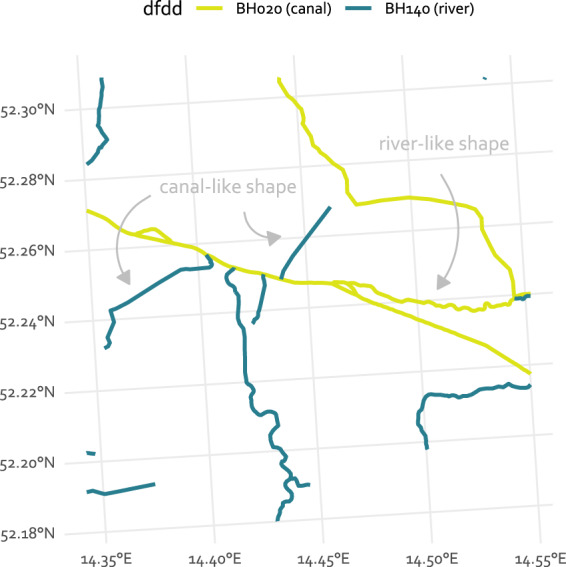
Example of the river network data showing the confusion between the values BH140 (river), BH020 (canal) and BH030 (ditch) of the attribute column dfdd of the river network dataset^15^.

As the generation of this dataset is based on the “EU-Hydro–River Network Database”, its accuracy and validity depends strongly on the quality of this underlying dataset. The “EU-Hydro–River Network Database”^15^ has been generated through a combination of photo interpretation of very high resolution imagery and drainage modelling based on the EU DEM with 25 m resolution. It comprises a river network for all of the EEA39 states at a high resolution. According to our research, there is no comprehensive quality assessment or validation of the version used. The visual inspection reveals some errors relevant to the methodology presented here. Firstly, a confusion of the classification of the linestring geometries into canals, ditches and rivers occurs frequently. An example for such confusion is shown in Fig. 12. Here, some relatively straight shaped linestring geometries are classified as river (value BH140 in column *dfdd*), whereas meandering geometries are classified as canal (value BH020 in column *dfdd*). Other errors might be introduced through the limitation of the spatial resolution of the photo imagery and the EU DEM. This potentially affects the detection and of smaller rivers, canals and ditches.

As previously mentioned in River Network, additional errors were found in the river network data. These errors relate to incorrect values in the *longpath* and *object_id* columns and are corrected in places where the resulting maps revealed incorrect patterns by visual inspection. These patterns were evident from the lack of a river network in larger regions. It is very likely that more errors of such type will remain in the river network with minor impact on the resulting maps. Fixing these errors programmatically requires a solid theoretical knowledge of processing networks and could be done in future versions of the “EU-Hydro - River Network Database”^15^.

### Administrative borders over land

The accuracy of this dataset may also be reduced near the boundaries that run over land rather than along the coast or river basin boundaries. This includes the regions that are close to the borders in the South and East of Turkey, in the East of continental Europe and in the East of Finland (see yellowish lines in Fig. 7). Here, the boundaries of the underlying dataset, and thus this dataset, follow administrative borders instead of river basin boundaries Therefore, calculated distances to the nearest stream in these regions may be inaccurate because another stream not included in the dataset could be closer to a raster cell center. The width of these potentially inaccurate regions along the margins increases with hydrologic order. Because the stream locations of adjacent stream networks are unknown, it is not possible to delineate this region or quantify its width. To address this issue when applying this dataset to such a region, a conservative option would be to truncate or mask these regions by shifting the corresponding boundaries inward by the maximum value in the stream distance map of the respective hydrologic order.

### Calculation of DD

**Fig. 13:**
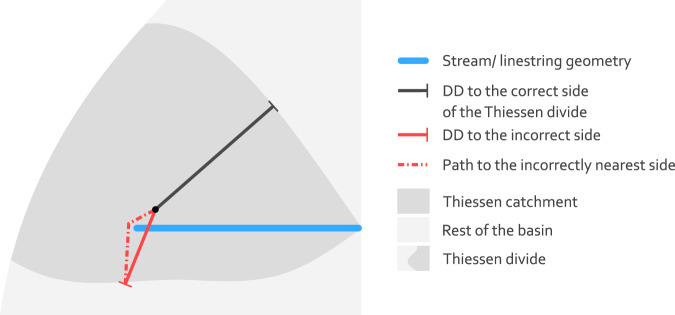
Schematic example showing the source of inaccurate of DD in areas near headwaters caused by the applied method to calculate DD. The red distance as DD is incorrect, because it crosses the stream and therefore does not fulfill the defined condition. The correct DD would be the dark grey distance. The path to the correct side is equal to the correct DD (dark grey solid line) and therefore not drawn on the schematic map.

Another inaccuracy is introduced by the method to calculate DD. This inaccuracy only affects a narrow area near headwaters. To calculate DD, the GRASS GIS command r.walk is used. The command r.walk originally aims at a different purpose than the one it is used for here. It calculates the cumulative costs for moving between two geographic locations based on topographic map and a map that represents friction costs. By increasing the cost parameters, it calculates the horizontal distance from a cell to the nearest Thiessen divide, preferring a path without crossing a stream. This behavior is usually achieved everywhere except for areas near headwaters where “walking” around the stream becomes an option. To illustrate this, following case is considered. If a linestring geometry representing a stream is closer to one side of the Thiessen divide than to the other side, r.walk calculates an incorrect distance around the start of the linestring as it cheaper to “walk” around the stream than walking a straight path from the more distant but correct side of the Thiessen divide. Thus, the straight path from this mistakenly nearest side of the Thiessen divide crosses the stream. The required and correct behaviour would be to calculate the distance as the length of a straight line to the Thiessen divide that does not cross the stream (Fig. 13).

### Surface water bodies in DSD Maps

The method for calculating DD also causes missing values (NA) for grid cells that are located inside larger surface water bodies such as lakes. This issue only affects the measure DSD or its related raster maps (“*<abbreviation of the EU-MOHP measure>* = *dsd*”). If required, a potential solution to this could be to fill these NA cells with values from the nearest non-NA grid cell as a simple approximation.

As stated below, we encourage readers and users of this dataset to report errors in the methods or code in the mentioned Github repository.

## Usage Notes

This data publication mainly provides two resources to be used by the research community. Firstly, the dataset itself and secondly, the source code to be adapted and applied to custom river network data. The former can be used as additional, hydrological context describing features in any machine learning or non-machine-learning-based modelling task in the domain of hydrology and hydrogeology across several scales. After downloading the required compressed *0.7z*-files from Hydroshare (see data-recordsData Records for download link), they can be decompressed using the free and open-source file archiver program 7-Zip. Due to the widely used GeoTIFF file format, the dataset can be processed and visualized through any GIS Software. For reasons of reproducibility in science, it is recommended to use programming languages instead of point-and-click software such as ArcGIS or QGIS. The programming languages R or Python provide a variety of tools to import, process and visualize GeoTIFF data but also offer flexibility from a machine learning perspective. The R packages *raster* and *stars* cover most common operations on raster data^31,32^. To crop the GeoTIFF files to your custom study area or area of interest, the function st_crop() from the *stars* package offers a fast cropping without having to read the large GeoTIFF files into memory. To do so, it’s required to read in the GeoTIFF files as *stars_proxy* objects with read_stars(<path to GeoTIFF file>, proxy = TRUE) before applying st_crop(). To simplify some of the previous steps, we developed the R package *eumohpclipr* (https://github.com/MxNl/eumohpclipr/)^33^. This package provides functionality to mosaic, crop or clip and plot the EU-MOHP dataset^20^. For a fast raster cell value extraction based on polygons, the R package *exactextractr* (https://github.com/isciences/exactextractr)^34^ is recommended.

It is important to note that raster cell values of all GeoTIFF files are stored as integers in the *INT32* data type to reduce storage size. Cell values of files that represent LP (“*<abbreviation of the EU-MOHP measure> = lp*”) must be divided by 100 to obtain percentages with two decimal digits or by 10,000 to obtain values in the range from 0 to 1. The cell values of all other files represent a distance in meters and can be used as is. All files are stored using the coordinate reference system (CRS) ETRS89-extended/LAEA Europe with the EPSG code 3035.

The following paragraphs focus on the usage of the source code for reproducing the EU-MOHP dataset^20^ and to use it for other custom datasets. They also provide information on the hardware and software setup as well as on major steps before getting the source code to to run.

The computations to generate the presented dataset^20^ were performed on a DELL PowerEdge C4140 Server with an Intel Xeon Gold 6240 R CPU and 384 GB installed RAM. The installed operating system is Microsoft Windows Server 2019 Standard, version 10.0.17763 Build 17763. The total runtime of the pipeline as well as of individual targets is summarised in Table 4.

**Table 4:** Overview of the runtime and data size of all targets or processing steps related to the generation of this dataset in descending order.

Target name	Runtime				Data size
	Seconds	Minutes	Hours	Days	Mb
db_objects_to_grass	1199656.6	19994.3	333.2	13.9	0.0
rivernetworks_merged_per_streamorder	155852.7	2597.5	43.3	1.8	2002.6
eumohp_files_compression	127353.4	2122.6	35.4	1.5	0.0
db_inland_waters_strahler	1869.6	31.2	0.5	0.0	0.0
river_basins_unioned	1296.2	21.6	0.4	0.0	87.8
coastline_unioned	653.4	10.9	0.2	0.0	84.2
coastline_buffer	586.0	9.8	0.2	0.0	7.7
river_basins_subset_union_in_db	472.5	7.9	0.1	0.0	76.0
coastline_filtered	363.3	6.1	0.1	0.0	76.5
river_networks	235.3	3.9	0.1	0.0	1112.0
db_river_networks_merged_per_streamorder	835.3	13.9	0.2	0.0	0.0
db_river_networks_clean	129.9	2.2	0.0	0.0	0.0
inland_waters	101.4	1.7	0.0	0.0	183.0
db_river_networks_strahler_studyarea	41.3	0.7	0.0	0.0	0.0
river_networks_clean	38.0	0.6	0.0	0.0	1051.0
db_inland_waters	25.5	0.4	0.0	0.0	0.0
river_networks_non_dry_selected_streamtypes	24.9	0.4	0.0	0.0	1053.7
rivernetworks_feature_id	126.3	2.1	0.0	0.0	2070.1
river_basins	20.3	0.3	0.0	0.0	147.3
river_basins_subset	8.7	0.1	0.0	0.0	84.0
streamorders	5.7	0.1	0.0	0.0	0.0
coastline_grouped	5.4	0.1	0.0	0.0	92.8
config.	4.8	0.1	0.0	0.0	0.0
filepath_coastline	4.8	0.1	0.0	0.0	0.0
studyarea_as_coastline	4.5	0.1	0.0	0.0	28.1
directory_river_networks	4.5	0.1	0.0	0.0	0.0
coastline_watershed	4.2	0.1	0.0	0.0	29.8
db_selected_studyarea	3.5	0.1	0.0	0.0	0.0
coastline_buffer_unioned	3.1	0.1	0.0	0.0	7.5
selected_studyarea	2.0	0.0	0.0	0.0	29.8
major_path_ids	1.9	0.0	0.0	0.0	4.4
bracket_start_ids	1.7	0.0	0.0	0.0	0.0
river_basins_region_name	1.7	0.0	0.0	0.0	29.8
distinct_streamorders_in_riverbasins	1.1	0.0	0.0	0.0	0.0
river_networks_imputed_streamorder_canals_as_1	0.1	0.0	0.0	0.0	1053.7
rivernetworks_merged_per_streamorder_grouped	0.0	0.0	0.0	0.0	2003.1
river_networks_files	0.0	0.0	0.0	0.0	0.0
river_basins_grouped	0.0	0.0	0.0	0.0	147.4
river_basins_files	0.0	0.0	0.0	0.0	0.0
river_basin_names	0.0	0.0	0.0	0.0	0.0
coastline_regrouped	0.0	0.0	0.0	0.0	84.2
river_networks_clip	0.0	0.0	0.0	0.0	1112.0
**Total**	**1489739.6**	**24829.1**	**413.7**	**17.2**	**12658.5**

The used software comprises R (version 4.0.3)^23^, PostgreSQL (version 13) database with the PostGIS (version 3.1.0) extension and GRASS GIS (version 7.8.5-2). R package dependencies are managed with the *renv* package^35^. The versions of used R packages can be found in the *renv.lock* file. Most used R packages are also listed in the references^24,29–32,35–55^.

**Fig. 14:**
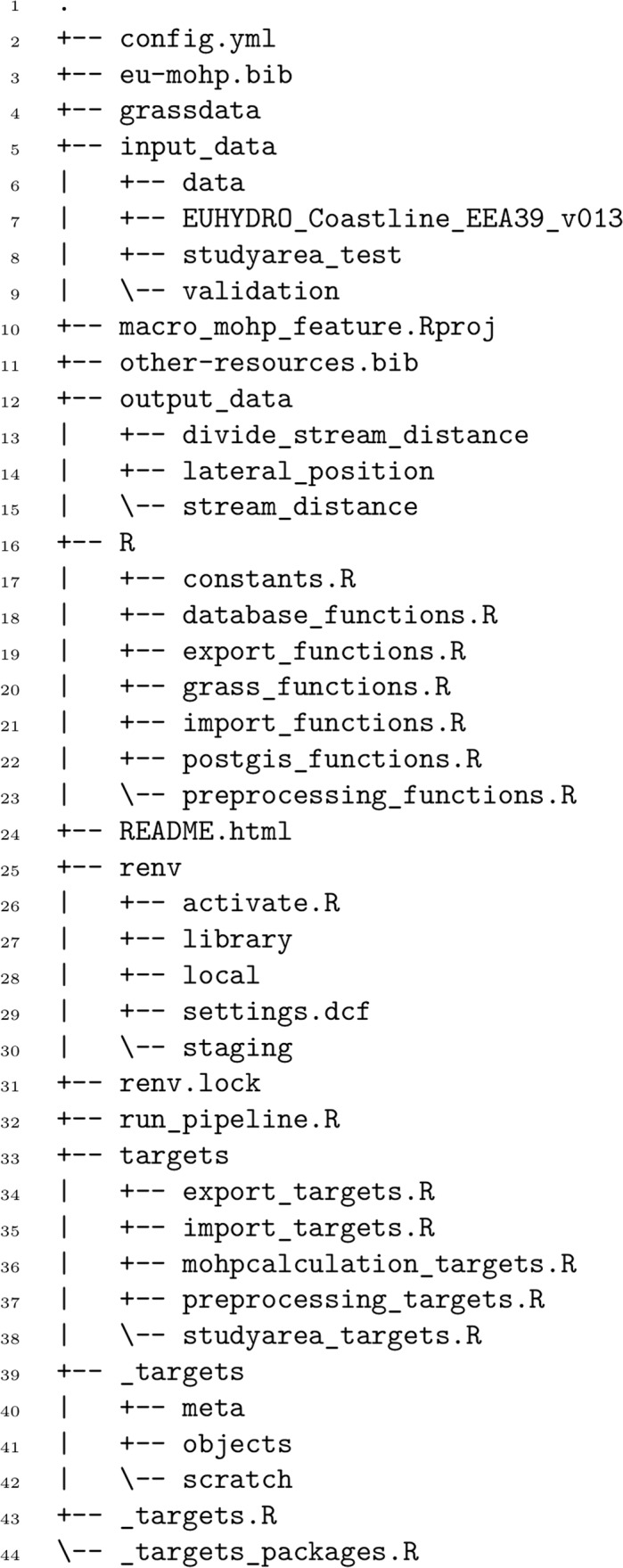
Directory tree of the project directory; only relevant subdirectories and files are listed here.

The directory and file structure of the project folder containing all code and files to generate this dataset is summarized in Fig. 14 in a tree structure. Files and directories that are not relevant for describing the methods are not shown here. The project folder as the top level directory is the working directory. The file *config.yml* (line 2) contains definitions of variables that are meant to be set by a user before running the *targets* pipeline. The most relevant variable is *cellsize* which sets the spatial resolution of the resulting EU-MOHP gridded maps^20^. Another important variable is *area* to switch between a test study area and the complete study area for all EEA39. The test study area represents a small fraction of the study area. This reduces the runtime of the pipeline for testing purposes. The folder *grassdata* (line 4) is used for writing the GRASS GIS databases to. The folder *input_data* (line 5) contains all required input data. Firstly, the sub-folder *data* (line 6) comprises the river network data as one single folder per basin as it is derived after unzipping the downloaded “EU-Hydro–River Network Database” data^15^ (see Underlying Dataset). The second sub-folder *EUHYDRO_Coastline_EEA39_v013* (line 7) contains the coastline data (see Underlying Dataset). The third sub-folder *studyarea_test* (line 8) contains a test study area as Shape file for pipeline testing purposes only (see Code availability). Lastly, the sub-folder *validation* contains all data required to calculate the values and figures for the cross-comparison in Technical Validation. The file *macro_mohp_feature.Rproj* (line 10) is the R project file. The folder *output_data* (line 12) contains three sub-directories where the final EU-MOHP gridded maps^20^ are written to. These directories are created by the pipeline if they don’t already exist. *R* (line 16) contains R scripts where custom functions and constants are defined. *renv* (line 25) and the file *renv.lock* (line 31) are related to the R package *renv* that tracks versions of package dependencies^35^. The R script *run_pipeline.R* (line 32) contains code to execute the *targets* pipeline that does all the data processing and calculations. *targets* (line 33) contains the definition of all targets or processing steps of the pipeline. For overview reasons, it is split thematically across multiple files. *_targets* (line 39) is used by the *targets* package internally. The file *_targets.R* (line 43) sets up the targets pipeline and loads all dependencies.

To reproduce this dataset, the subsequent steps are required. They have been tested under Windows as operating system (see above in this section), therefore deviations under Linux or MacOS are likely:

1. Install the R language, PostgreSQL, PostGIS and GRASS GIS in their previously described versions. Furthermore, install the latest version of RStudio. RStudio is a free integrated development environment for R.
2. Set up a PostgreSQL database with the name “postgis” or, alternatively, choose a different name and change the variable *database_name* in the *config.yml* file later. Independently from the database name, change the setting of the PostgreSQL database to not request a password for connection.
3. Download the project repository containing all required code and scripts from the above mentioned static code repository.
4. Download the required input data “EU-Hydro–River Network Database”^15^ and “EU-Hydro–Coastline”^22^ from the links below and store it in the directory *input_data* as described previously to make it match the file structure of the *input_data* (Fig. 14, line 5–8). For downloading the data a free user account is required. Alternatively, if you want to keep the data at another directory, e.g. on a remote server, you need to change the file paths in the file *constants.R*.
5. Navigate to the project directory and open the file *macro_mohp_feature.Rproj* with RStudio.
6. Install the package *renv* by running following command in the R-console
  install.packages (“renv”)
7. Install all package dependencies with the subsequent line in the R-console. Note that under Linux and MacOS some R-packages have system dependencies, such as the package sf, which depends on libgeos-dev, among others. Please consult the respective documentation when facing an issue.
  renv:: restore ()
8. Before running the pipeline on the full spatial coverage of the EEA39 countries, we recommend to test the pipeline with the smaller test study area by setting the variable *area* in the file *config.yml* to “test”. The runtime will be around 20 min. The content of the *config.yml* should look like this (Note the empty line in line 6):
  area: testcellsize: 30database_name: postgisexclude_scandinavian_basins: FALSEsimplify_polygons: FALSEdata_descriptor_only: FALSEparallel: TRUE
  If the pipeline works in “test” mode, you can change the variable *area* back to “europe”.
9. Start the processing pipeline by running the file *run_pipeline.R* from an R-console and in the root directory with
  source (“run_pipeline.R”)
  or, alternatively, from the command line with
  Rscript run_pipeline.R
10. If you encounter any problems, please contact the corresponding author or preferably open a Github issue. Errors can probably be caused by incorrect directories and file paths. If the available memory is insufficient, one option is to run the pipeline sequentially rather than in parallel. To do this, change the variable *parallel* in the file *config.yml* from TRUE to FALSE.
11. To reproduce the data descriptor itself, you can execute the pipeline after a successful run by setting the variable *data_descriptor_only* in the file *config.yml* to “TRUE”.

The required underlying datasets “EU-Hydro–River Network Database”^15^ version v013 can be downloaded from the Copernicus Land Monitoring Service (https://land.copernicus.eu/imagery-in-situ/eu-hydro/eu-hydro-river-network-database?tab=download) as well as the “EU-Hydro–Coastline”^22^ version v013 (https://land.copernicus.eu/imagery-in-situ/eu-hydro/eu-hydro-coastline?tab=download). In order to maximize and simplify reproducibility, we currently plan to set up a docker container. For availability updates, please visit the mentioned Github repository. For transferring the presented methods to another custom region, equivalent input data to Table 1 is required.

## Data Availability

As stated previously, all processing steps including the generation of the dataset, most of the figures and the manuscript are script based. All required source code^56^ can be found on Hydroshare (10.4211/hs.8ea376970c904c6698fc8cfe392689de) as a static code repository. Due to the procedure of the reviewing process, this static code repository only contains the status of the code before the last reviewing iteration. The final code used for submitting the reviewed manuscript can be found in this separate code release on Github (https://github.com/MxNl/macro_mohp_feature/releases/tag/v013.1.1.0). The actively developed code can be also found in the same repository on Github (https://github.com/MxNl/macro_mohp_feature). We encourage interested users of this dataset to report errors in the code or to give hints on further methodological or programming improvements through opening an issue in the Github repository or contacting the corresponding author via E-mail^13,55^.
